# Evaluation of 2 Ultraviolet-C Light Boxes for Decontamination of N95 Respirators

**DOI:** 10.20411/pai.v6i1.432

**Published:** 2021-06-05

**Authors:** Jennifer L. Cadnum, Basya S. Pearlmutter, Daniel F. Li, Annette L. Jencson, Jacob G. Scott, Ian C. Charnas, Curtis J. Donskey

**Affiliations:** 1 Research Service, Louis Stokes Cleveland VA Medical Center, Cleveland, Ohio; 2 Cleveland Clinic Lerner Research Institute, Cleveland, Ohio; 3 Case Western Reserve University School of Medicine, Cleveland, Ohio; 4 Case Western Reserve University School of Engineering and Sears think[box], Cleveland, Ohio; 5 Geriatric Research, Education, and Clinical Center, Louis Stokes Cleveland VA Medical Center, Cleveland, Ohio

**Keywords:** ultraviolet light, bacteriophage MS2, N95 respirator, SARS-CoV-2

## Abstract

**Background:**

Ultraviolet-C (UV-C) light devices are effective in reducing contamination on N95 filtering facepiece respirators. However, limited information is available on whether UV-C devices meet the Food and Drug Administration's (FDA) microbiological requirements for Emergency Use Authorization (EUA) for respirator bioburden reduction.

**Methods:**

We tested the ability of 2 UV-C light boxes to achieve the 3-log_10_ microorganism reductions required for EUA for reuse by single users. Whole 3M 1860 or Moldex 1513 respirators were inoculated on the exterior facepiece, interior facepiece, and internal fibers with bacteriophage MS2 and/or 4 strains of bacteria and treated with UV-C cycles of 1 or 20 minutes. Colorimetric indicators were used to assess penetration of UV-C through the respirators.

**Results:**

For 1 UV-C box, a 20-minute treatment achieved the required bioburden reduction for Moldex 1513 but not 3M 1860 respirators. For the second UV-C box, a 1-minute treatment achieved the required bioburden reduction in 4 bacterial strains for the Moldex 1513 respirator. Colorimetric indicators demonstrated penetration of UV-C through all layers of the Moldex 1513 respirator but not the 3M 1860 respirator.

**Conclusions:**

Our findings demonstrate that UV-C box technologies can achieve bioburden reductions required by the FDA for EUA for single users but highlight the potential for variable efficacy for different types of respirators.

## INTRODUCTION

Decontamination and reuse of N95 respirators is not recommended but may be considered in crisis situations such as shortages encountered during the coronavirus disease 2019 (COVID-19) pandemic [1]. Several technologies using vaporized hydrogen peroxide or steam have received emergency use authorization (EUA) for respirator decontamination from the Food and Drug Administration (FDA) [2]. These technologies require relatively long treatment cycles and transfer to a central processing area. Because fit performance may decrease with repeated donning and doffing [3], the Centers for Disease Control and Prevention (CDC) recommends a maximum of 4 bioburden reduction cycles per respirator or 5 donnings, whichever comes first [1]. However, a recent study suggested that N95 respirators may remain effective after extensive reuse with the caveat that users should consistently perform a seal check and obtain a good a seal before donning a reused N95 [4].

A recent systematic review suggested that ultraviolet-C (UV-C) light might be the most practical method currently available for respirator decontamination [5]. UV-C light technologies are widely used in healthcare facilities and could provide relatively rapid decontamination of respirators at the point of care [5, 6]. Some healthcare systems have implemented use of UV-C for decontamination of respirators that are reused by individuals [7]. However, UV-C may have reduced efficacy against organisms associated with irregular or soft surfaces, including the straps or interior surfaces of some respirators [5–7]. Several studies have demonstrated that efficacy of UV-C may vary for different models of respirator and different material types [8, 9].

One important consideration for healthcare facilities is whether technologies used for respirator decontamination meet the FDA criteria for EUA [2, 10]. For decontamination of respirators for single-users to supplement existing CDC reuse recommendations (tier 3), demonstration of >3-log10 reductions of either a non-enveloped virus or 2 gram-positive and 2 gram-negative bacteria is required [10]. Currently, only 1 UV-box device has received an EUA for respirator bioburden reduction [2, 11]. For this device, the FDA approval specifies that the device does not sterilize or decontaminate respirators, but only provides bioburden reduction. Moreover, it is specified that the approval was only for the 3M 1860 model and the respirators being treated must be placed in a breathable paper bag and held for a minimum of 5 days prior to UV-C treatment [11]. Here, we tested 2 UV-C light boxes to determine if they would meet the FDA-tier 3 requirements for decontamination of 2 types of respirators [2].

## METHODS

### Test organisms

The test organisms included bacteriophage MS2 (American Type Culture Collection [ATCC] 15597-B1), methicillin-resistant *Staphylococcus aureus* (MRSA) (ATCC 43300), vancomycin-resistant *Enterococcus faecium* VanB-type strain C68 (VRE), *Escherichia coli* (ATCC 15597), and *Klebsiella pneumoniae* (ATTC 700603). Bacteriophage MS2 was propagated in Escherichia coli as described previously [3].

### Decontamination devices tested

**Figure 1.**
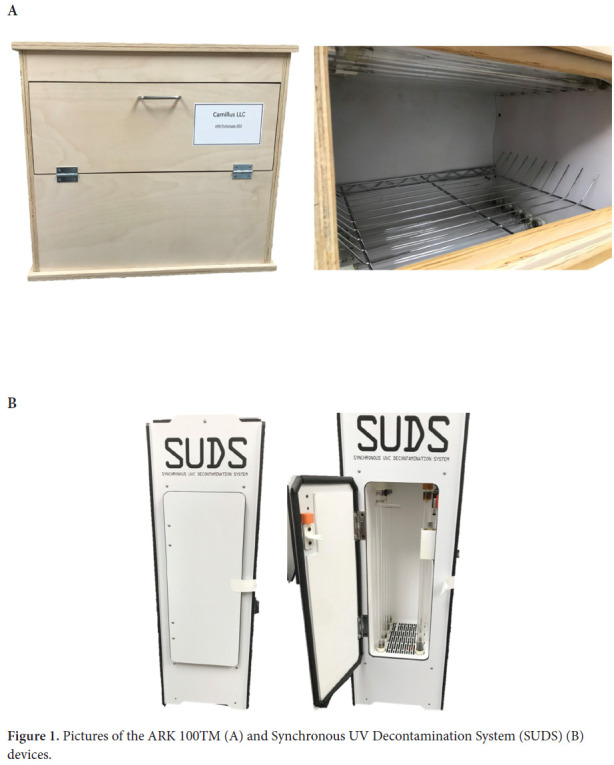
Pictures of the ARK 100TM (A) and Synchronous UV Decontamination System (SUDS) (B) devices.

Figure 1 provides pictures of the prototypes of the 2 study devices used for testing. The ARK 100^TM^ (Camillus LLC, Cleveland, Ohio) UV-C box has a decontamination chamber size of 21x16x23 inches with 8 low-pressure mercury lamps below and 8 above the items to be decontaminated. Per the manufacturer, measurements of UV-C irradiance at a central location inside the UV-C box demonstrated that a 10-minute cycle delivers a dose of >6 J/cm2. The Synchronous UV Decontamination System (SUDS) is a compact UV-C box designed for rapid point-of-care decontamination of single N95 respirators [7]. The device has 8 high output low-pressure mercury UV-C bulbs that deliver UV-C in close proximity to all surfaces of a respirator. Based on measurements of UV-C irradiance at all points within the SUDS system, a 1-minute cycle delivers a dose of at least 2 J/cm2, regardless of the position within the device [7].

### Decontamination test protocol

**Figure 2.**
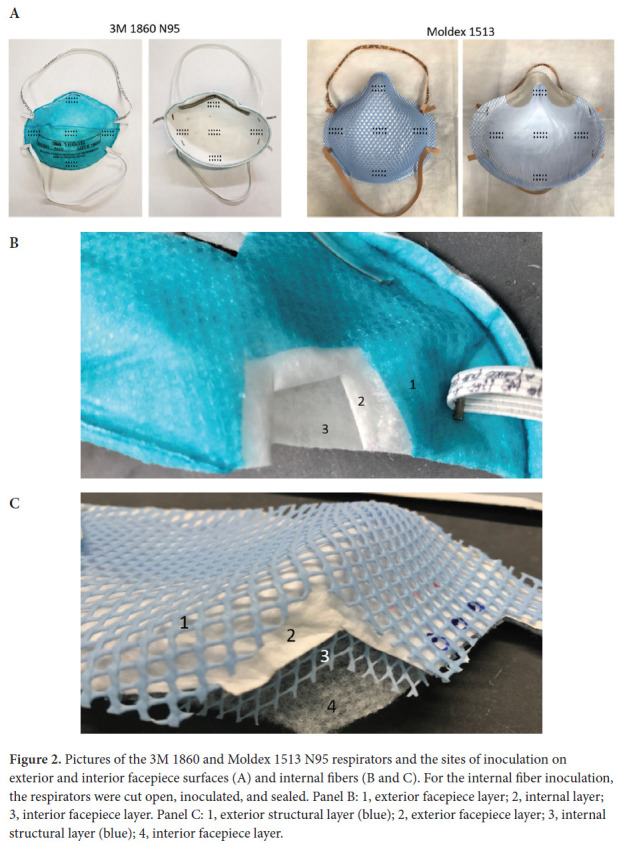
Pictures of the 3M 1860 and Moldex 1513 N95 respirators and the sites of inoculation on exterior and interior facepiece surfaces (A) and internal fibers (B and C). For the internal fiber inoculation, the respirators were cut open, inoculated, and sealed. Panel B: 1, exterior facepiece layer; 2, internal layer; 3, interior facepiece layer. Panel C: 1, exterior structural layer (blue); 2, exterior facepiece layer; 3, internal structural layer (blue); 4, interior facepiece layer.

The current FDA guidance for testing of respirator bioburden reduction systems does not specify required sites of inoculation [10]. Therefore, we chose sites of inoculation based on discussions with FDA scientists; it was recommended that whole respirators should be tested, that 5 specific sites should be included, and that testing should include the exterior surface, interior surface, and internal fibers of the respirators. Whole 3M 1860 (3M; Saint Paul, MN) and Moldex 1513 (Mold-ex-Metric, Inc; Culver City, CA) respirators were inoculated in triplicate on exterior facepiece surfaces, interior surfaces, and internal fibers at 5 locations each including the center and edges of the facepiece (Figure 2A). Separate respirators were used for exterior, interior, and internal fiber inoculation. For the internal fiber inoculation, the respirators were cut open, inoculated, and sealed (Figure 2B and 2C).

For the ARK UV-C box, initial testing was conducted with bacteriophage MS2. If results for a respirator did not pass criteria for tier 3 with bacteriophage MS2, additional testing was conducted using the bacteria. For the ARK UV-C box, criteria for tier 3 with bacteriophage MS2 were passed for the Moldex 1513 respirator but not the 3M 1860; therefore, testing with bacteria was only conducted with the 3M 1860 respirator.

For the SUDS UV-C box, previous testing demonstrated that criteria for tier 3 with bacteriophage MS2 was not passed for the Moldex 1513 respirator or 3M 1860 respirator [7]. In addition, preliminary experiments demonstrated that the SUDS device did not reduce MRSA by greater than 3 log10 after inoculation into the interior mask or the internal fibers of the 3M 1860 respirator. Therefore, for the current study, we tested whether the SUDS device would meet microbiologic criteria for tier 3 for the Moldex 1513 respirator when testing was conducted using the 4 strains of bacteria.

Ten droplets of 1 µL containing a total of ~10^6^ colony-forming units (CFU) or plaque-forming units (PFU) of the test organisms were applied in 1-cm2 areas and allowed to air dry; this method of inoculation was chosen because it is a standard test method recommended for determining antimicrobial efficacy of UV-C light against influenza virus on fabric carriers [12]. The organisms were suspended in 50% Artificial Saliva Soiling Agent [12]. Single respirators were placed in the center of the box and treated for 20 minutes (ARK) or 1 minute (SUDS); the 20-minute cycle for the ARK device was chosen because preliminary experiments demonstrated that the 10-minute cycle recommended by the manufacturer did not meet tier 3 requirements for reduction in bacteriophage MS2 [10]. After treatment, inoculated sections were cut out and processed to quantify viable organisms [5]. All tests were performed in triplicate. Log10 PFU or CFU reductions were calculated by comparing recovery from treated versus untreated respirators. A mean reduction of 3 log10 or greater was considered effective [2, 6].

Colorimetric indicators (UVC 100 Dosimeter Cards; Intellego Technologies AB, Gothenburg, Sweden) were used to assess UV-C delivery by the ARK box [13]. The indicators are yellow in the absence of UV-C exposure but change to orange and pink when exposed to UV-C doses of approximately 50 and 100 mJ/cm^2^, respectively. According to the manufacturer, the 50 and 100 mJ/cm^2^ doses have been shown to be adequate to kill vegetative bacteria and *Clostridioides difficile* spores, respectively. To assess penetration of UV-C through the full thickness of respirator material, indicators were placed against the interior or exterior facepiece during UV-C cycles.

## RESULTS

**Figure 3.**
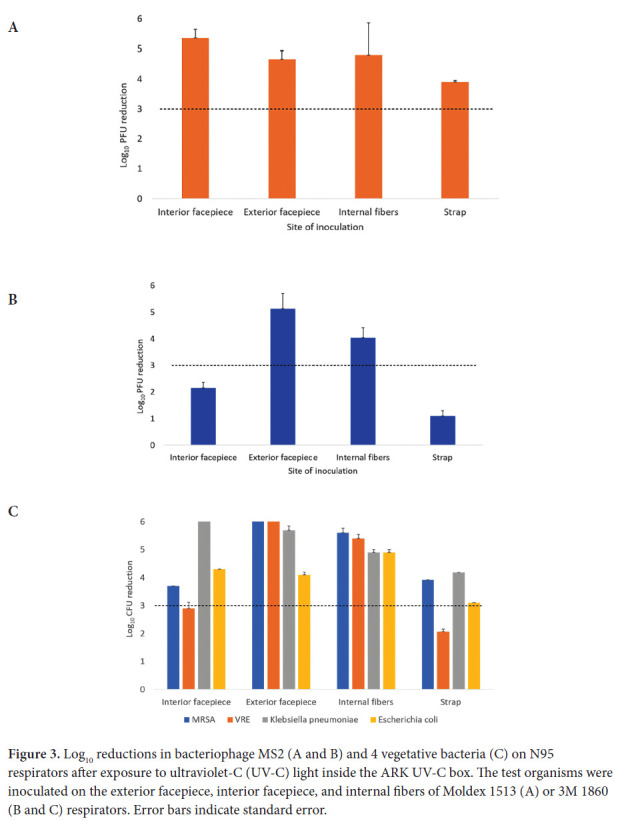
Log_10_ reductions in bacteriophage MS2 (A and B) and 4 vegetative bacteria (C) on N95 respirators after exposure to ultraviolet-C (UV-C) light inside the ARK UV-C box. The test organisms were inoculated on the exterior facepiece, interior facepiece, and internal fibers of Moldex 1513 (A) or 3M 1860 (B and C) respirators. Error bars indicate standard error.

A 20-minute treatment cycle in the ARK UV-C box reduced bacteriophage MS2 by greater than 3 log_10_ at all sites on the Moldex 1513 respirator (Figure 3A). For the 3M 1860 respirator, a 20-minute treatment reduced bacteriophage MS2 by greater than 3 log10 on the exterior facepiece and internal fibers of the 3M 1860 respirator, but not on the interior facepiece (Figure 3B). Twenty-minute cycles in the ARK UV-C box reduced all 4 bacterial strains by greater than 3 log10 on the exterior facepiece and internal fibers of the 3M 1860 respirator, but not the internal facepiece where the VRE strain was reduced by a mean of 2.9 log10 CFU while the other strains were reduced by greater than 3 log10 (Figure 3C). The exterior surfaces of the 3M 1860 were impermeable whereas the liquid suspensions were absorbed into the interior surface material.

**Figure 4.**
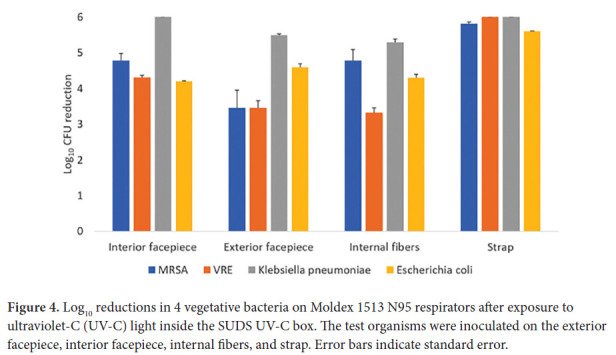
Log_10_ reductions in 4 vegetative bacteria on Moldex 1513 N95 respirators after exposure to ultraviolet-C (UV-C) light inside the SUDS UV-C box. The test organisms were inoculated on the exterior facepiece, interior facepiece, internal fibers, and strap. Error bars indicate standard error.

**Figure 5.**
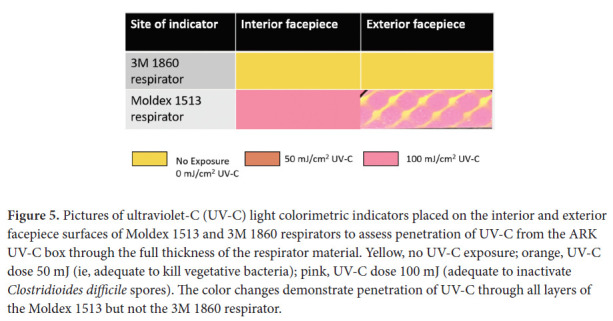
Pictures of ultraviolet-C (UV-C) light colorimetric indicators placed on the interior and exterior facepiece surfaces of Moldex 1513 and 3M 1860 respirators to assess penetration of UV-C from the ARK UV-C box through the full thickness of the respirator material. Yellow, no UV-C exposure; orange, UV-C dose 50 mJ (ie, adequate to kill vegetative bacteria); pink, UV-C dose 100 mJ (adequate to inactivate *Clostridioides difficile* spores). The color changes demonstrate penetration of UV-C through all layers of the Moldex 1513 but not the 3M 1860 respirator.

A 1-minute treatment cycle in the SUDS UV-C box reduced each of the 4 bacteria by greater than 3 log_10_ at all sites on the Moldex 1513 respirator (Figure 4). Colorimetric indicators placed on the interior and exterior surfaces of the respirators demonstrated penetration of a UV-C dose of >100 mJ/cm^2^ through all layers of the Moldex 1513, with the exception of some blockage matching the pattern of the outer blue structural layer only when the indicator was applied directly onto the structural layer (Figure 5). For the 3M 1860 respirator, UV-C did not change the color of the indicators demonstrating that UV-C was unable to penetrate through all layers of respirator material.

## DISCUSSION

Emergency use authorization from the FDA provides assurance that technologies proposed for respirator decontamination or bioburden reduction are effective while maintaining safety and performance [2, 9]. A 20-minute treatment in the ARK UV-C box met tier 3 microbiologic criteria for the Moldex 1513 respirator based on reduction of the non-enveloped virus bacteriophage MS2 and nearly met tier 3 criteria for the 3M 1860 respirator based on testing with 4 bacterial organisms (ie, only VRE was not reduced by >3 log_10_). A 1-minute treatment with the SUDS device met tier 3 criteria for the Moldex 1513 respirator based on reduction of the 4 bacterial species but did not meet tier 3 criteria for the 3M 1860 respirator. These findings demonstrate that some UV-C box technologies can meet tier 3 criteria for respirator bioburden reduction but also highlight the need to conduct testing of each brand of respirator that will be decontaminated by UV-C.

Previous studies have demonstrated reduced efficacy of UV-C light on the internal facepiece and strap surfaces of some respirators [5–8]. The 3M 1860 respirator has a permeable interior facepiece lining that absorbs the liquid inoculum. Based on the colorimetric indicator results, the layers of the 3M 1860 respirator prevent full penetration of UV-C light, whereas UV-C penetrates through all layers of the Moldex 1513 respirator. Although the FDA does not recommend testing the strap [9], the 3M 1860 strap also absorbs the inoculum and has reduced log10 reductions.

One potential concern regarding testing of UV-C technologies for N95 respirator bioburden reduction is the applicability of the laboratory test protocols to real-world situations. The testing protocol we used involved applying a relatively large inoculum to exterior and interior surfaces and internal fibers. The exterior facepiece of the respirator is generally considered the surface that presents the highest risk for pathogen transfer to the wearer [9]. The significance of virus particles absorbed below the external surfaces of respirators is unclear. Experimental evidence suggests that re-aerosolization of virus particles from contaminated respirators is negligible [9, 14]. Thus, protocols that involve application of virus only to the exterior facepiece may more closely simulate real-world contamination. In a previous study, the SUDS device reduced MS2 applied as a 1-mL inoculum to the entire exterior facepiece of a respirator by greater than 3 log10 when only the surface was sampled [6].

Our study has some limitations. Only 2 UV-C technologies were tested. However, in preliminary experiments, similar results were obtained with a UVDI-360 Room Sanitizer (Ultraviolet Devices, Inc) positioned 8 inches from the respirator surfaces. Because bacteriophage MS2 is relatively resistant to UV-C light [5], additional studies are needed with enveloped viruses including severe acute respiratory syndrome coronavirus 2 (SARS-CoV-2). Finally, we did not evaluate the impact of the UV-C treatment on factors such as filtration and fit. Some previous studies have suggested that high UV-C doses may alter the strength of respirator materials, including weakening of the straps [15, 16]. However, testing conducted by the National Personal Protective Technology demonstrated that 20 cycles of UV-C treatment with the SUDS device did not adversely affect filtration efficiency and manikin fit [15].
